# Black Tea High-Molecular-Weight Polyphenol-Rich Fraction Promotes Hypertrophy during Functional Overload in Mice

**DOI:** 10.3390/molecules22040548

**Published:** 2017-03-29

**Authors:** Yuki Aoki, Tetsuo Ozawa, Tohru Takemasa, Osamu Numata

**Affiliations:** 1 Graduate School of Life and Environmental Sciences, University of Tsukuba, 1-1-1 Tennodai, Tsukuba, Ibaraki 305-8572, Japan; yuki.aoki87@gmail.com (Y.A.); tetsuoozawa@ybb.ne.jp (T.O.); 2 Graduate School of Comprehensive Human Sciences, University of Tsukuba, 1-1-1 Tennodai, Tsukuba, Ibaraki 305-8574, Japan; takemasa.tohru.gm@u.tsukuba.ac.jp

**Keywords:** black tea, polyphenol, overload, hypertrophy, Akt, mTOR

## Abstract

Mitochondria activation factor (MAF) is a high-molecular-weight polyphenol extracted from black tea that stimulates training-induced 5′ adenosine monophosphate-activated protein kinase (AMPK) activation and improves endurance capacity. Originally, MAF was purified from black tea using butanol and acetone, making it unsuitable for food preparation. Hence, we extracted a MAF-rich sample “E80” from black tea, using ethanol and water only. Here, we examined the effects of E80 on resistance training. Eight-week old C57BL/6 mice were fed with a normal diet or a diet containing 0.5% E80 for 4, 7 and 14 days under conditions of functional overload. It was found that E80 administration promoted overload-induced hypertrophy and induced phosphorylation of the Akt/mammalian target of rapamycin (mTOR) pathway proteins, such as Akt, P70 ribosomal protein S6 kinase (p70S6K), and S6 in the plantaris muscle. Therefore, functional overload and E80 administration accelerated mTOR signaling and increased protein synthesis in the muscle, thereby inducing hypertrophy.

## 1. Introduction

The history of tea is very long. People in Asia have been consuming tea for about 4000 years [1]. Recently, many reports showed that low-molecular-weight tea components, such as caffeine, polyphenols, and catechins, have various effects at both individual and cellular levels. Teas have beneficial physiological effects in antioxidation [2], anti-obesity [3,4], and acceleration of metabolism [5,6]. They also play a role in the prevention of disuse skeletal muscle atrophy [7,8]. Above all, tea polyphenols inhibit prostate carcinogenesis and reduce the risk of breast cancer recurrence [9,10].

Skeletal muscle is highly adaptive and responds well to environmental and physiological stimulation [11]. There are two types of skeletal muscle fibers, slow and fast, which exhibit different characteristics. Slow-type fibers can continually contract for hours, whereas fast-type fibers have a higher contractile velocity but a lower resistance to fatigue [12]. Training causes a striking fiber type shift in the skeletal muscle [13]. For example, endurance training causes a fiber type shift from fast to slow muscle fibers, which improves the aerobic capacity of the muscle. On the other hand, resistance training causes a shift from slow to fast muscle fibers, which improves the central nervous system’s ability to recruit muscle and increases muscle mass and strength [14,15]. Therefore, resistance training induces muscle hypertrophy characterized by an increase in the size and number of muscle fibers. Skeletal muscle hypertrophy is controlled by protein synthesis. The main mediator of protein synthesis in the skeletal muscle is considered to be the insulin-like growth factor-1 (IGF-1)/phosphoinositide 3-kinase (PI3K)/Akt pathway, wherein Akt promotes the activation of two independent signaling pathways, mTOR and glycogen synthase kinase 3β (GSK3β) [15].

We previously reported a high-molecular-weight polyphenol derived from black tea that enhanced the mitochondrial membrane potential of the ciliated protozoan *Tetrahymena pyriformis* [16], and named it mitochondria activation factor (MAF). In addition, mice administered with 0.04% MAF combined with seven weeks of training (15 m/min for 30 min, five days per week) showed a significant improvement in endurance capacity and could run a longer distance for a longer period compared with the exercise only group [17]. In a previous report, green tea polyphenols prevented disuse skeletal muscle atrophy in aged or sarcopenic rodents [7,8]. These results indicate that tea polyphenols play a role in maintaining muscle mass or preventing muscle atrophy. However, the role of polyphenols in black tea has not yet been reported.

Previously, we purified MAF from black tea using butanol and acetone, which was unsuitable for food preparation. Hence, in this paper, we developed a safer method for the preparation of a MAF-rich fraction “E80” from black tea, using only ethanol and water. To test whether E80 promotes overload-induced hypertrophy and activates mTOR signaling in the skeletal muscle, we induced functional overload in mice with or without administration of E80. Several parameters indicative of overload-induced hypertrophy were assessed. Our data demonstrated that E80 activates intracellular signaling pathways that involve mTOR while also promoting overload-induced hypertrophy.

## 2. Results

### 2.1. Composition of E80

**Figure 1:**
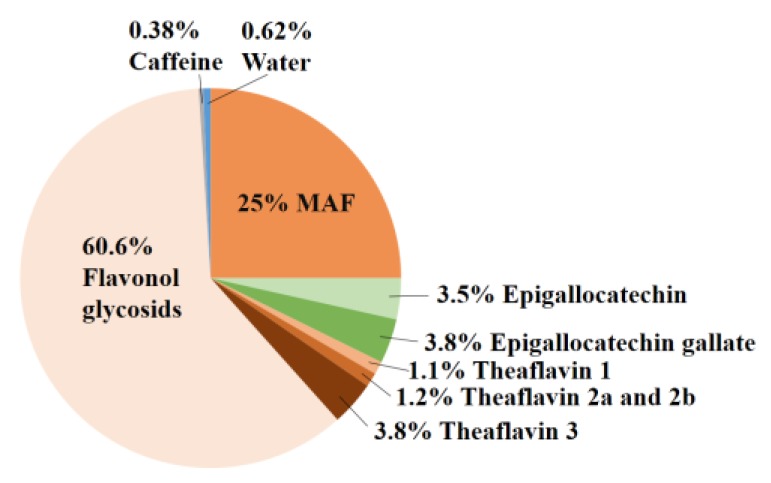
Components of E80. E80 included 25% mitochondria activation factor (MAF), 3.5% epigallocatechin, 3.8% epigallocatechin gallate, 1.1% theaflavin 1, 1.2% theaflavin 2a and 2b, 3.8% theaflavin 3, 60.6% flavonol glycosides, 0.62% water and 0.38% caffeine.

Previously, MAF was purified from black tea using *n*-butanol and acetone. Since that was unsuitable for food preparation [18], we sought another approach to purify MAF. Extraction of a MAF-rich fraction from black tea was successfully achieved with ethanol and water using Toyopearl HW-40F. We named this fraction “E80” since it was eluted with 80% ethanol. The components of E80 were 25% MAF, 3.5% epigallocatechin, 3.8% epigallocatechin gallate, 1.1% theaflavin-1, 1.2% theaflavin-2a and -2b, 3.8% theaflavin-3, 60.6% flavonol glycosides, 0.62% water and 0.38% caffeine (Figure 1). A small amount of caffeine (0.38%) is an important constituent of E80. Because a large dose of caffeine induces severe symptoms [19], tea extract containing caffeine is sometimes unsuitable for use as a supplement.

### 2.2. E80 Has No Effect on Food Intake and Growth

**Figure 2:**
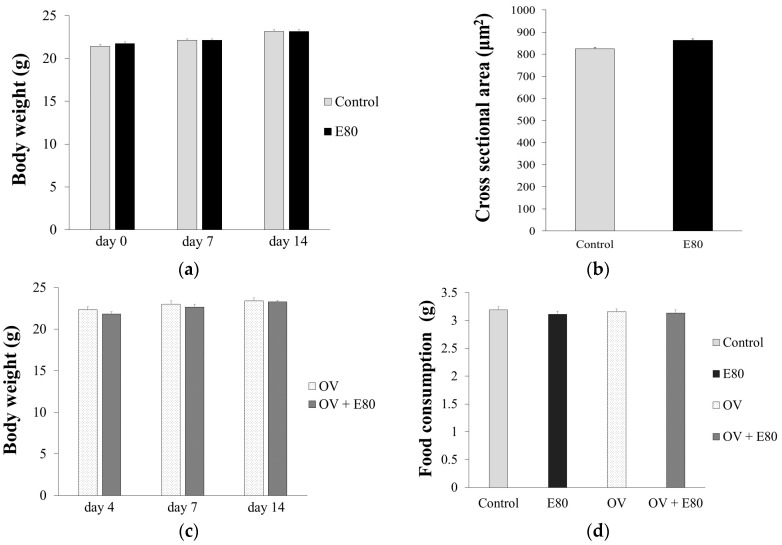
E80 has no effect on food intake and growth. (**a**,**b**) Body weight change (**a**) and CSA (cross sectional area) of plantaris muscle (**b**) at day 14 in the control and E80 groups; (**c**) Body weight change at day 14 in the overload (OV) and OV + E80 groups; (**d**) Food consumption per day in the control, E80, OV, and OV + E80 groups. Values are presented as mean ± SEM (*n* = 8). SEM, standard error of mean.

The body weight of the control group was almost the same as that of the E80 group after 14 days (Figure 2a, Table 1). Cross sectional area (CSA) of the plantaris muscle of the control group was almost the same as that of the E80 group on day 14 (Figure 2b, Table 1). In addition, after overload surgery, there was no difference in body weight or food consumption between the overload (OV) and OV + E80 groups after 14 days (Figure 2c,d, Table 1). These data show that E80 intake alone does not affect food consumption, body growth, or skeletal muscle mass.

### 2.3. E80 Improves Overload-Induced Muscle Hypertrophy

**Figure 3:**
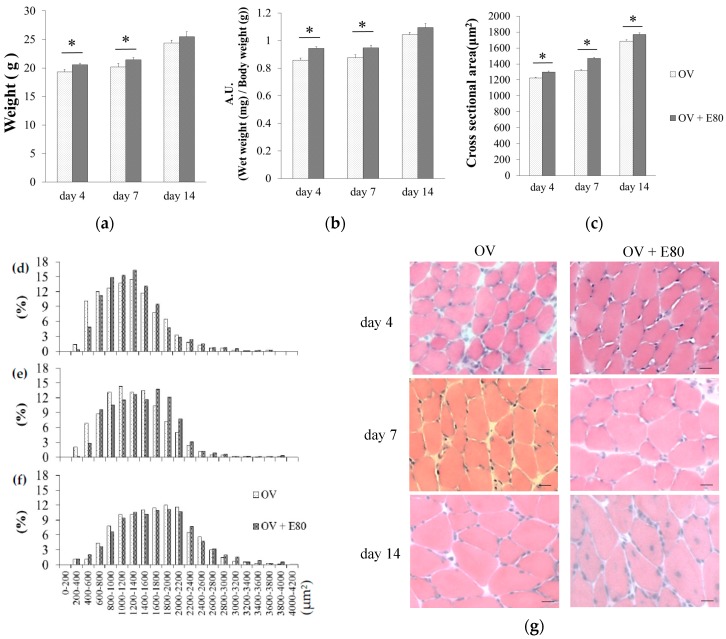
E80 improves overload-induced muscle hypertrophy. (**a**–**c**) Muscle weight (**a**), muscle wet weight/body weight (**b**), and CSA (cross sectional area) (**c**) of the plantaris muscle in the OV and OV + E80 groups; (**d**–**f**) Distribution of plantaris muscle fibers in the OV and OV + E80 groups on days 4, 7 and 14; (**g**) Representative images of hematoxylin and eosin (H&E)-stained plantaris muscle. Values are presented as mean ± SEM. Scale bars are 20 µm. * indicates significant difference (*p* < 0.05) (*n* = 8). SEM, standard error of mean.

To examine the effect of E80 on overload-induced hypertrophy, we compared the wet weight of plantaris muscle and the wet weight of plantaris muscle/body weight and CSA of plantaris muscle between the OV and OV + E80 groups, on day 4 and day 7. The wet weight of plantaris muscle of the OV + E80 group was significantly higher than that of the OV group (Figure 3a). The wet weight of plantaris muscle/body weight of the OV + E80 group was also higher than that of the OV group (Figure 3b). As for CSA, the fibers of the OV + E80 group were much larger than those of the OV group on days 4, 7, and 14 (Figure 3c–g). Especially on day 7, CSA of the OV + E80 group was 12% larger than that of the OV group (Figure 3c). This difference can be seen in the pictures (Figure 3g). We also measured the frequency distribution of CSA and compared it between the OV + E80 and OV groups (Figure 3d–f). We observed a moderate rightward shift in the frequency distribution of CSA of the OV + E80 group on day 4 and day 7 (Figure 3d–f), but this shift disappeared on day 14 (Figure 3f). The rightward shift was consistent with the difference in the wet weight of plantaris muscle and the wet weight of plantaris muscle/body weight between the OV + E80 and OV groups on day 4 and day 7. The reason for dissipation of shift at day 14 was that functional overload-induced hypertrophy was reaching convergence point. These data show that the OV + E80 group has thicker muscle fibers than the OV group.

### 2.4. E80 Intake Activates the Akt/mTOR Signaling Pathway

**Figure 4:**
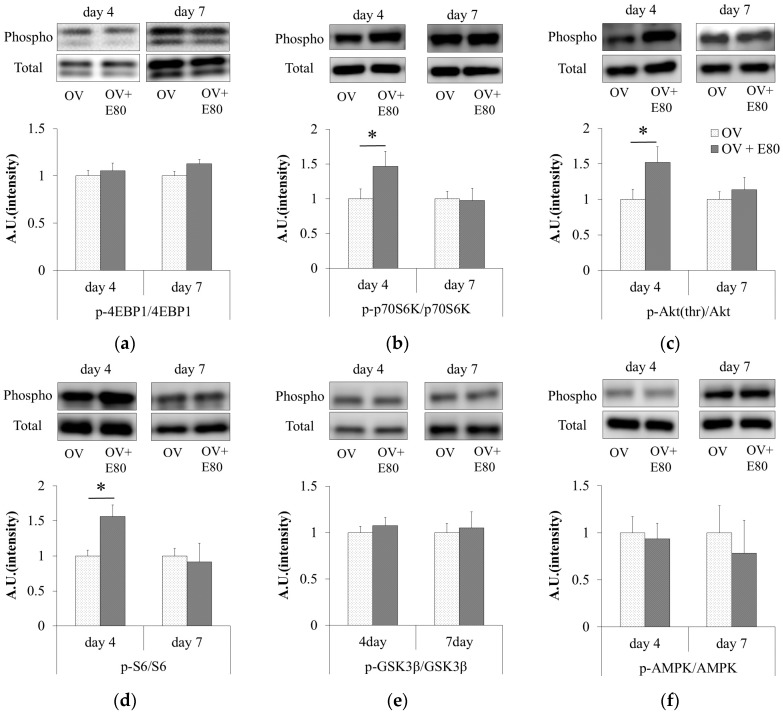
E80 intake activates the Akt/mTOR signaling pathway. (**a**–**f**) Western blot bands of total and phosphorylated proteins (above) and relative ratio of phosphorylated protein/total protein (below) in the plantaris muscle using antibodies against p-Akt(thr) and Akt (**a**); p-p70S6K and p70S6K (**b**); p-4EBP1 and 4EBP1 (**c**); p-S6 and S6 (**d**); p-GSK3β and GSK3β (**e**); and p-AMPK (adenosine monophosphate-activated protein kinase) and AMPK (**f**). Quantitative data represent mean ± SEM. * indicates significant difference (*p* < 0.05) (*n* = 8). SEM, standard error of mean.

We measured the level of Akt phosphorylation in the plantaris muscle of functional overload mice, and compared it between the OV + E80 and OV groups. On day 4, the phosphorylation level of Akt in the plantaris muscle of the OV + E80 group significantly increased (Figure 4a). Next, we evaluated the phosphorylation levels of p70S6K and 4EBP1. On day 4, the phosphorylation level of p70S6K significantly increased in the plantaris muscle of the OV + E80 group (Figure 4b), but that of 4EBP1 did not increase (Figure 4c). Since S6 is a direct target of p70S6K [14], the phosphorylation level of S6 was measured. Consistent with that of p70S6K, the phosphorylation level of S6 increased on day 4 in the plantaris muscle of the OV + E80 group (Figure 4d). On the other hand, no change was observed in the phosphorylation levels of GSK3β and AMPK (Figure 4e,f). These data show that E80 intake promotes the phosphorylation of Akt, p70S6K, and S6 in the plantaris muscle of functional overload mice on day 4.

This figure shows the signaling pathways regulated by IGF/mTOR pathway.

## 3. Discussion

In this study, we observed that supplementation of E80 promotes the phosphorylation of Akt, p70S6K, and S6 in functional overload mice (Figure 4a,b,d). Activation of these molecules are crucial indicators of muscle hypertrophy [15]. Consistent with these data, an increase in muscle mass and CSA were observed in the OV + E80 group compared with the OV group (Figure 3a–c,g).

**Figure 5:**
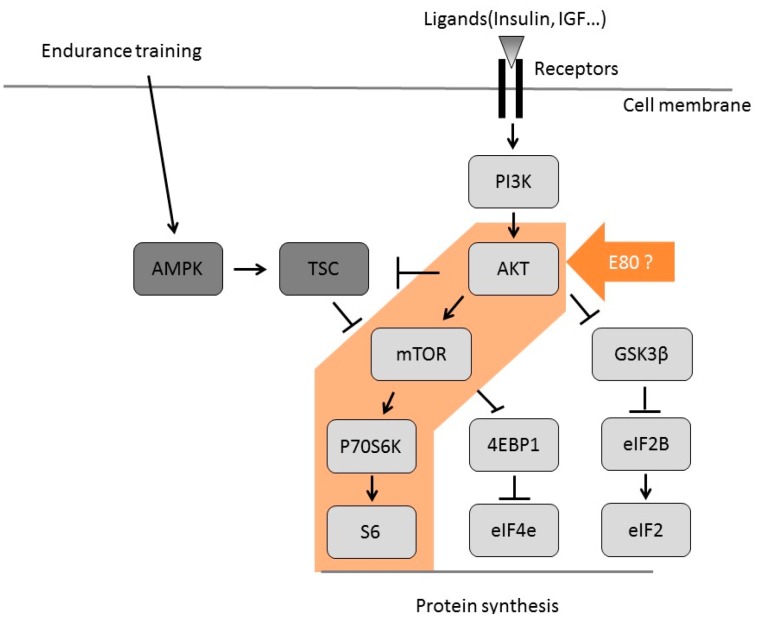
Signaling cascade of protein synthesis in skeletal muscle.

Skeletal muscle hypertrophy and an increase in muscle mass and CSA were thought to be a result of increased protein synthesis. Skeletal muscle protein synthesis is regulated by the IGF/mTOR pathway [15] (Figure 5). Akt promotes protein synthesis in the skeletal muscle, and its function is regulated by phosphorylation [15]. Moreover, Akt promotes the activation of mTOR and its downstream targets, such as p70S6K and Eukaryotic translation initiation factor 4E-binding protein 1 (4EBP1) [15]. During endurance training, AMPK is activated and it directly phosphorylates TSC2 (Tuberous sclerosis complex2) [15]. This leads to inhibition of mTOR, suggesting that endurance training inhibits protein synthesis.

Akt functions as a crucial regulator of skeletal muscle hypertrophy. Akt promotes the activation of GSK3β and mTOR via independent pathways (Figure 5), which is important for skeletal muscle hypertrophy [20]. When GSK3β is phosphorylated, the phosphorylation of Eukaryotic Initiation Factor 2 (eIF2B) on serine 535 decreases and the translation initiation and protein synthesis process is promoted [15]. The levels of phosphorylated p70S6K correlate with the magnitude of skeletal muscle hypertrophy after resistance exercise [20]. In addition, the degree of p70S6K and S6 phosphorylation in human skeletal muscle depends on the training volume [21]. These reports suggest that elevated phosphorylation of p70S6K (indicator of mTOR activity) is essential for skeletal muscle hypertrophy. In this experiment, E80 intake promoted the phosphorylation of Akt, p70S6K, and S6 (Figure 4a,b,d), suggesting that E80 activates Akt and the downstream targets of mTOR (Figure 5). E80 was thought to regulate protein synthesis via the Akt and S6 signaling pathways, and thereby promote overload-induced skeletal muscle hypertrophy. Hence, E80 can be considered a useful supplement for increasing skeletal muscle hypertrophy and may have a potential role in human skeletal muscle development

AMPK activates Peroxisome proliferator-activated receptor gamma coactivator 1-alpha (PGC1-α), which regulates mitochondria biogenesis [22,23]. In addition, AMPK is a key regulator of mitochondrial oxidative capacity, skeletal muscle metabolism, and endurance performance [23,24,25]. With regard to hypertrophy induced by functional overload, there are some inconsistent reports on the role of AMPK. Some groups have reported that AMPK decreases mTOR activity in vitro [26,27,28,29]. In addition, AMPK-knockout has been shown to result in greater overload-induced hypertrophy [30]. On the other hand, other groups have reported that AMPK is activated by functional overload [31]. In our previous work, we reported that MAF intake with endurance exercise significantly stimulates exercise training-induced improvement of endurance capacity in mice via the association between AMPK and GLUT4 [17]. In this experiment, E80 had no effect on AMPK activity under conditions of functional overload (Figure 4f). Due to the composition of E80, E80 and MAF may have different effects on skeletal muscle subjected to both functional overload and endurance exercise. Therefore, the effects of E80 on skeletal muscle subjected to endurance exercise must be examined. If E80 administration in combination with endurance exercise improves endurance capacity, it suggests that E80 has two physiological roles, improving endurance and increasing muscle mass.

Many athletes use supplements to improve their training efficiency. As mentioned above, training for resistance and endurance cannot be achieved at the same time. However, both are necessary for almost all kinds of sports. E80 has the potential to be an ideal supplement for many athletes, because it effectively influences both resistance and endurance exercise. Moreover, it is difficult to activate p70S6K in older men with resistance training. p70S6K phosphorylation increases the rate of protein synthesis in young men but not in older men [31]. These reports suggest that it is difficult for older men to activate signal transmission in the cell and to increase protein synthesis during resistance exercise. The main cause of sarcopenia is thought to be the gradual loss of muscle mass and function [32,33]. If E80 promotes p70S6K phosphorylation and protein synthesis in aged people, it may be an ideal supplement for gaining muscle mass and preventing function loss caused by sarcopenia. In summary, E80 can be used as a supplement for both older men and athletes. However, further studies to assess the effect of E80 on humans are important.

## 4. Materials and Methods

### 4.1. Mice and Functional Overload

All animal procedures were performed in accordance with the institutional guidelines for the care and use of laboratory animals as approved by the University of Tsukuba. Animals were subjected to functional overload according to a previously described method [34]. Using mice anesthetized with 2–3% isoflurane, under aseptic conditions, we made a small incision in the posterior lower limb, exposed a part of the ankle extensor muscles and Achilles tendon, and removed the entire soleus and gastrocnemius muscles, resulting in functional overload of the remaining plantaris muscle. After removal of the two muscles, we closed the skin incision with adhesive agents. This procedure was applied for both hind limbs. On days 4, 7 and 14 after surgery, the mice were anesthetized with isoflurane and subsequently sacrificed. The plantaris muscle was carefully dissected and wet weights were determined immediately. They were then frozen in liquid nitrogen and stored at −80 °C until use.

### 4.2. Preparation of MAF-Rich Sample (E80) from Black Tea

Black tea (30 g; Daily club; Mitsui Norin Co., Ltd., Tokyo, Japan) was brewed for 1 min in boiling water (1000 mL), and then allowed to stand for 10 min. The brew was filtered using double-layered filter paper (number 2; Advantec, Toyo Roshi Kaisha, Tokyo, Japan). The filtrate was mixed with 250 mL Toyopearl HW-40F (Tosoh, Tokyo, Japan) previously washed with water. After 30 min, Toyopearl HW-40F was filtered using a filter paper and washed with 150 mL of water 10 times. Polyphenolic substances adsorbed to Toyopearl were extracted 12 times with 150 mL of 80% (*v/v*) warm ethanol. All extracts were evaporated under reduced pressure and freeze dried to yield 2.96 g. This dark brown amorphous powder was named E80.

### 4.3. Quantitative Analysis of Caffeine, Catechins, and Theaflavins in E80

High performance liquid chromatography (HPLC) analysis was performed using the Inertsil ODS-3 column (4.6 unit × 250 mm, 5 µm; GL Sciences, Tokyo, Japan) connected to a Shimadzu Class M10A HPLC system (Shimadzu, Kyoto, Japan). The column was eluted at 40 °C with a linear gradient from 5% to 40% (*v/v*) acetonitrile containing 0.02% (*v/v*) trifluoroacetic acid over 70 min at a flow rate of 0.7 mL/min and monitored for absorbance at 280 nm. Quantitative determination was based on the calibration curve prepared with an adequate concentration of a pure commercial standard. Samples were dissolved in 10% (*v/v*) acetonitrile and 4.0 µL portions were injected into the column.

### 4.4. Quantitative Analysis of Highly Polymerized Polyphenols in E80

For preparation of the analytical sample from E80, 382 mg E80 was dissolved in water (100 mL). The solution was extracted eight times with 40 mL ethyl acetate to remove the ethyl acetate-soluble constituents. The water phase was evaporated under reduced pressure to remove the ethyl acetate in the solution and the pH was adjusted to three with hydrochloric acid. The solution was then extracted five times with 40 mL *n*-butanol. The aqueous phase of *n*-butanol was concentrated under reduced pressure at 50 °C and freeze dried. The yield was 140 mg.

For quantitative analysis of the highly polymerized polyphenols in E80, we used the following analytical instruments that consisted of two medium-pressure SP-11 delivery pumps (Tokyo Rika Kikai, Tokyo, Japan): a gradient mixer, a sample injector VI-II (EYELA), a medium pressure glass column (1 unit × 30 cm) packed with Toyopearl HW-40F, and a fraction collector CHF161RA (Advantec, Tokyo, Japan). Analytical sample (1.7 mL) dissolved in 20% (*v/v*) acetone was injected into the column, which had been conditioned with 20% acetone. The column was eluted using a linear gradient containing from 20% to 50% acetone (total volume 180 mL) at a flow rate of 0.4 mL/min. The eluent (1.5 g) was collected by a fraction collector and the absorbency was measured at 350 nm. From the elution profile, the highly polymerized polyphenol fractions were combined, evaporated under reduced pressure, freeze dried, and then weighed.

### 4.5. Preparing Diet

Mice in the normal diet group were fed with a powdered diet (NMF; Oriental yeast Co., Tokyo, Japan) only, and those in E80 group were fed with NMF mixed with 0.5% E80. Four groups were prepared: functional overload mice eating only NMF (OV group), functional overload mice eating NMF mixed with E80 (OV + E80 group), non-treated mice eating only NMF (control group), and non-treated mice eating NMF mixed with E80 (E80 group). All groups were free to drink water and eat food.

### 4.6. Antibodies

We used the following rabbit antibodies purchased from Cell Signaling Technology (Danvers, MA, USA): anti-Akt (No. 9272), anti-phospho-Akt (Ser473; No. 4060), anti-p70S6K (No. 9202), anti-phospho-p70S6K (Thr389; No. 9205), anti-S6 (No. 2217), anti-phospho-S6 (Ser235/236; No. 4858), anti-4EBP1 (No. 9644), anti-phospho-4EBP1 (Thr37/46; No. 2855), anti-GSK3β (No. 12456), anti-phospho-GSK3β (Ser9; No. 5558), anti-AMPK (No. 2532), anti-phospho-AMPK (Ser9; No. 2535), and anti-rabbit IgG (No. 7074).

### 4.7. Cross-Sectional Area Quantification

The plantaris muscle was covered in optimal cutting temperature (OCT) compound (Sakura Finetek, Tokyo, Japan), and then quickly frozen in liquid nitrogen-cooled isopentane and stored at −20 °C until sectioning. Frozen muscles were sectioned at thickness 7 μm, air dried, and stored at −20 °C. Images were captured with the Olympus BX-51 microscope (Tokyo, Japan).

To determine the CSA of muscle fibers, the muscle sections were incubated with Mayer’s hematoxylin solution (Wako, Osaka, Japan) for five min to stain the nuclei and then washed with water for one min. Following this, they were stained with eosin solution (Wako, Osaka, Japan) for one min and then washed with water for one min. The stained sections were observed under the BX-51 microscope and CSA analysis was carried out using the Image J software.

### 4.8. Western Blot Analysis

To prepare total protein lysate, frozen muscle samples were homogenized in lysis buffer (1% Nonidet-P40, 1% sodium deoxycholate, 0.2% SDS, 150 mM NaCl, 50 mM 4-(2-hydroxyethyl)-1-piperazineethanesulfonic acid, 10 mM Ethylenediaminetetraacetic acid, 10 mM NAF, 10 mM Na_4_P_2_O_7_, and 2 mM Na_3_VO_4_) supplemented with 1% protease inhibitor cocktail for mammalian tissues (No. 162-0177; Nacalai Tesque, Kyoto, Japan). Protein concentration was determined by the the bicinchoninic acid method using the Protein Assay Bicinchoninate Kit (No. 06385-00, Nacalai Tesque, Kyoto, Japan). Protein extracts were electrophoresed on 7.5–12.5% acrylamide gels and subsequently transferred to polyvinylidene difluoride membranes (PVDF; No. 162-0177; Bio-Rad, Hercules, CA, USA). The membranes were blocked with 5% skim milk in Tris-buffered saline with Tween 20 (50 mM Tris, 138 mM NaCl, 2.7 mM KCl, and 0.05% Tween 20) for 45 min at room temperature (20~25 °C) and then incubated overnight with primary antibody at 4 °C followed by incubation with anti-rabbit IgG for 90 min. The band intensity was evaluated using the LI-COR system (No. CDG002134) and quantified by the Image Studio Digits 4.0 software (Nacalai Tesque, Kyoto, Japan).

### 4.9. Statistical Analysis

A one-way analysis of variance (ANOVA) was performed using the SPSS software (IBM Corp., New York, NY, USA) to determine whether a significant interaction exists between two independent factors.

## Figures and Tables

**Table 1:** E80 doesn’t affect food intake and growth. This table shows the values presented in Figure 2. ± indicates standard error of mean.

	Body Weight (g)			Food Consumption (g)	CSA (µm^2^)
	day 4	day 7	day 14		
Control	21.44 ± 0.36	22.13 ± 0.4	23.17 ± 0.37	3.19 ± 0.06	824.75 ± 5.44
E80	21.74 ± 0.31	22.14 ± 0.34	23.19 ± 0.15	3.11 ± 0.06	862.83 ± 6.23
OV	22.33 ± 0.38	22.99 ± 0.34	23.4 ± 0.36	3.15 ± 0.06	
OV + E80	21.8 ± 0.33	22.64 ± 0.35	23.27 ± 0.32	3.13 ± 0.06	

## Abbreviations

The following abbreviations are used in this manuscript:

4EBP1	Eukaryotic translation initiation factor 4E-binding protein 1
AMPK	5′ adenosine monophosphate-activated protein kinase
ANOVA	Analysis of variance
CSA	Cross sectional area
eIF2	Eukaryotic Initiation Factor 2
eIF2B	Eukaryotic Initiation Factor 2B
eIF4E	Eukaryotic Initiation Factor 4E
GSK3β	Glycogen synthase kinase 3 beta
HPLC	High performance liquid chromatography
IGF	Insulin-like growth factors
MAF	Mitochondria activation factor
mTOR	Mammalian target of rapamycin
p70S6K	P70 ribosomal protein S6 kinase
PGC1-α	Peroxisome proliferator-activated receptor gamma coactivator 1-alpha
PI3K	Phosphoinositide 3-kinase
PVDF	Polyvinylidene Difluoride
S6	Ribosomal protein S6 kinase
TSC	Tuberous sclerosis complex
